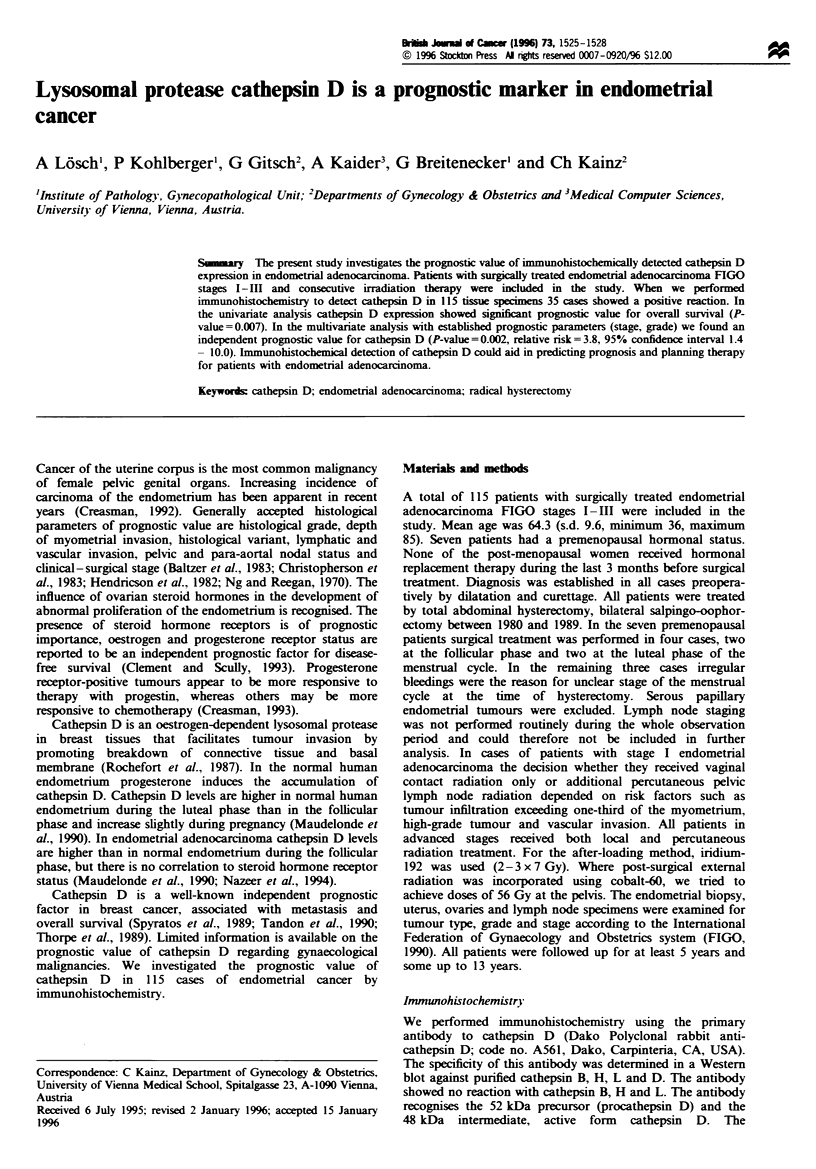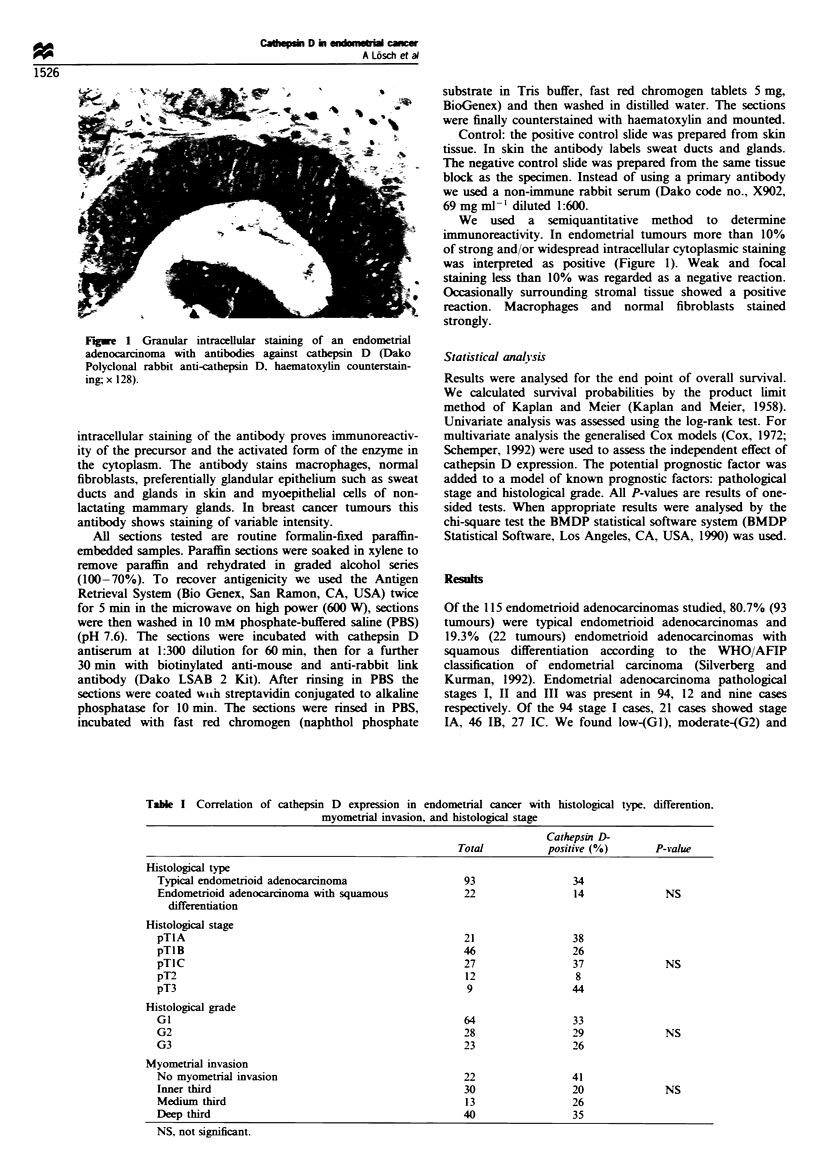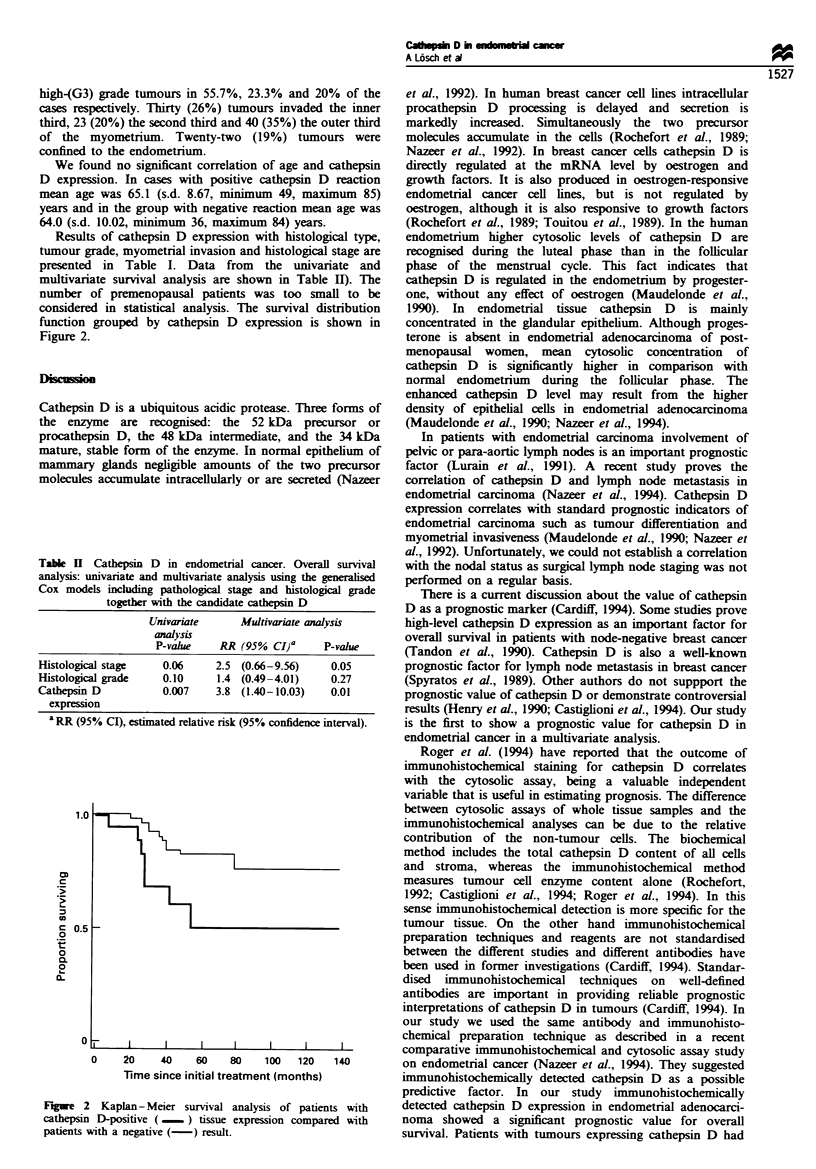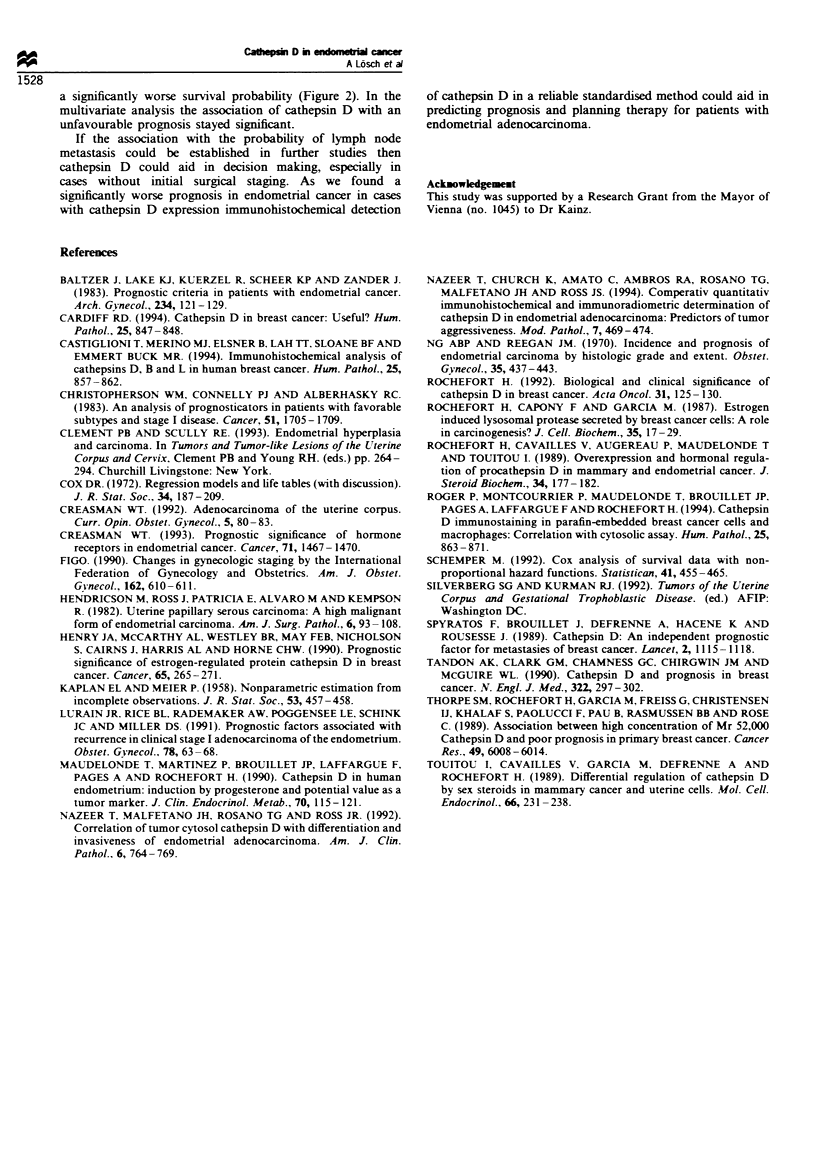# Lysosomal protease cathepsin D is a prognostic marker in endometrial cancer.

**DOI:** 10.1038/bjc.1996.287

**Published:** 1996-06

**Authors:** A. Lösch, P. Kohlberger, G. Gitsch, A. Kaider, G. Breitenecker, C. Kainz

**Affiliations:** Institute of Pathology, University of Vienna, Austria.

## Abstract

**Images:**


© 1996 Stockton Press All rights reserved 0007-0920/96 $12.00

### Lysosomal protease cathepsin D is a prognostic marker in endometrial cancer

A Lösch[1], P Kohlberger[1], G Gitsch[2], A Kaider[3], G Breitenecker[1] and Ch Kainz[2]

[1]*Institute of Pathology, Gynecopathological Unit;* [2]*Departments of Gynecology & Obstetrics and* [3]*Medical Computer Sciences, University of Vienna, Vienna, Austria.*

**Summary** The present study investigates the prognostic value of immunohistochemically detected cathepsin D expression in endometrial adenocarcinoma. Patients with surgically treated endometrial adenocarcinoma FIGO stages I–III and consecutive irradiation therapy were included in the study. When we performed immunohistochemistry to detect cathepsin D in 115 tissue specimens 35 cases showed a positive reaction. In the univariate analysis cathepsin D expression showed significant prognostic value for overall survival (*P*-value = 0.007). In the multivariate analysis with established prognostic parameters (stage, grade) we found an independent prognostic value for cathepsin D (*P*-value = 0.002, relative risk = 3.8, 95% confidence interval 1.4 – 10.0). Immunohistochemical detection of cathepsin D could aid in predicting prognosis and planning therapy for patients with endometrial adenocarcinoma.

**Keywords:** cathepsin D; endometrial adenocarcinoma; radical hysterectomy

Cancer of the uterine corpus is the most common malignancy of female pelvic genital organs. Increasing incidence of carcinoma of the endometrium has been apparent in recent years (Creasman, 1992). Generally accepted histological parameters of prognostic value are histological grade, depth of myometrial invasion, histological variant, lymphatic and vascular invasion, pelvic and para-aortal nodal status and clinical–surgical stage (Baltzer *et al.*, 1983; Christopherson *et al.*, 1983; Hendricson *et al.*, 1982; Ng and Reegan, 1970). The influence of ovarian steroid hormones in the development of abnormal proliferation of the endometrium is recognised. The presence of steroid hormone receptors is of prognostic importance, oestrogen and progesterone receptor status are reported to be an independent prognostic factor for disease-free survival (Clement and Scully, 1993). Progesterone receptor-positive tumours appear to be more responsive to therapy with progestin, whereas others may be more responsive to chemotherapy (Creasman, 1993).

Cathepsin D is an oestrogen-dependent lysosomal protease in breast tissues that facilitates tumour invasion by promoting breakdown of connective tissue and basal membrane (Rochefort *et al.*, 1987). In the normal human endometrium progesterone induces the accumulation of cathepsin D. Cathepsin D levels are higher in normal human endometrium during the luteal phase than in the follicular phase and increase slightly during pregnancy (Maudelonde *et al.*, 1990). In endometrial adenocarcinoma cathepsin D levels are higher than in normal endometrium during the follicular phase, but there is no correlation to steroid hormone receptor status (Maudelonde *et al.*, 1990; Nazeer *et al.*, 1994).

Cathepsin D is a well-known independent prognostic factor in breast cancer, associated with metastasis and overall survival (Spyratos *et al.*, 1989; Tandon *et al.*, 1990; Thorpe *et al.*, 1989). Limited information is available on the prognostic value of cathepsin D regarding gynaecological malignancies. We investigated the prognostic value of cathepsin D in 115 cases of endometrial cancer by immunohistochemistry.

Correspondence: C Kainz, Department of Gynecology & Obstetrics, University of Vienna Medical School, Spitalgasse 23, A-1090 Vienna, Austria
Received 6 July 1995; revised 2 January 1996; accepted 15 January 1996

### Materials and methods

A total of 115 patients with surgically treated endometrial adenocarcinoma FIGO stages I–III were included in the study. Mean age was 64.3 (s.d. 9.6, minimum 36, maximum 85). Seven patients had a premenopausal hormonal status. None of the post-menopausal women received hormonal replacement therapy during the last 3 months before surgical treatment. Diagnosis was established in all cases preoperatively by dilatation and curettage. All patients were treated by total abdominal hysterectomy, bilateral salpingo-oophorectomy between 1980 and 1989. In the seven premenopausal patients surgical treatment was performed in four cases, two at the follicular phase and two at the luteal phase of the menstrual cycle. In the remaining three cases irregular bleedings were the reason for unclear stage of the menstrual cycle at the time of hysterectomy. Serous papillary endometrial tumours were excluded. Lymph node staging was not performed routinely during the whole observation period and could therefore not be included in further analysis. In cases of patients with stage I endometrial adenocarcinoma the decision whether they received vaginal contact radiation only or additional percutaneous pelvic lymph node radiation depended on risk factors such as tumour infiltration exceeding one-third of the myometrium, high-grade tumour and vascular invasion. All patients in advanced stages received both local and percutaneous radiation treatment. For the after-loading method, iridium-192 was used (2–3 × 7 Gy). Where post-surgical external radiation was incorporated using cobalt-60, we tried to achieve doses of 56 Gy at the pelvis. The endometrial biopsy, uterus, ovaries and lymph node specimens were examined for tumour type, grade and stage according to the International Federation of Gynaecology and Obstetrics system (FIGO, 1990). All patients were followed up for at least 5 years and some up to 13 years.

### *Immunohistochemistry*

We performed immunohistochemistry using the primary antibody to cathepsin D (Dako Polyclonal rabbit anti-cathepsin D; code no. A561, Dako, Carpinteria, CA, USA). The specificity of this antibody was determined in a Western blot against purified cathepsin B, H, L and D. The antibody showed no reaction with cathepsin B, H and L. The antibody recognises the 52 kDa precursor (procathepsin D) and the 48 kDa intermediate, active form cathepsin D. The

**Figure 1** Granular intracellular staining of an endometrial adenocarcinoma with antibodies against cathepsin D (Dako Polyclonal rabbit anti-cathepsin D, haematoxylin counterstaining; × 128).

intracellular staining of the antibody proves immunoreactivity of the precursor and the activated form of the enzyme in the cytoplasm. The antibody stains macrophages, normal fibroblasts, preferentially glandular epithelium such as sweat ducts and glands in skin and myoepithelial cells of non-lactating mammary glands. In breast cancer tumours this antibody shows staining of variable intensity.

All sections tested are routine formalin-fixed paraffin-embedded samples. Paraffin sections were soaked in xylene to remove paraffin and rehydrated in graded alcohol series (100–70%). To recover antigenicity we used the Antigen Retrieval System (Bio Genex, San Ramon, CA, USA) twice for 5 min in the microwave on high power (600 W), sections were then washed in 10 mM phosphate-buffered saline (PBS) (pH 7.6). The sections were incubated with cathepsin D antiserum at 1:300 dilution for 60 min, then for a further 30 min with biotinylated anti-mouse and anti-rabbit link antibody (Dako LSAB 2 Kit). After rinsing in PBS the sections were coated with streptavidin conjugated to alkaline phosphatase for 10 min. The sections were rinsed in PBS, incubated with fast red chromogen (naphthol phosphate substrate in Tris buffer, fast red chromogen tablets 5 mg, BioGenex) and then washed in distilled water. The sections were finally counterstained with haematoxylin and mounted.

Control: the positive control slide was prepared from skin tissue. In skin the antibody labels sweat ducts and glands. The negative control slide was prepared from the same tissue block as the specimen. Instead of using a primary antibody we used a non-immune rabbit serum (Dako code no., X902, 69 mg $ml^{-1}$ diluted 1:600.

We used a semiquantitative method to determine immunoreactivity. In endometrial tumours more than 10% of strong and/or widespread intracellular cytoplasmic staining was interpreted as positive (Figure 1). Weak and focal staining less than 10% was regarded as a negative reaction. Occasionally surrounding stromal tissue showed a positive reaction. Macrophages and normal fibroblasts stained strongly.

### *Statistical analysis*

Results were analysed for the end point of overall survival. We calculated survival probabilities by the product limit method of Kaplan and Meier (Kaplan and Meier, 1958). Univariate analysis was assessed using the log-rank test. For multivariate analysis the generalised Cox models (Cox, 1972; Schemper, 1992) were used to assess the independent effect of cathepsin D expression. The potential prognostic factor was added to a model of known prognostic factors: pathological stage and histological grade. All *P*-values are results of one-sided tests. When appropriate results were analysed by the chi-square test the BMDP statistical software system (BMDP Statistical Software, Los Angeles, CA, USA, 1990) was used.

### Results

Of the 115 endometrioid adenocarcinomas studied, 80.7% (93 tumours) were typical endometrioid adenocarcinomas and 19.3% (22 tumours) endometrioid adenocarcinomas with squamous differentiation according to the WHO/AFIP classification of endometrial carcinoma (Silverberg and Kurman, 1992). Endometrial adenocarcinoma pathological stages I, II and III was present in 94, 12 and nine cases respectively. Of the 94 stage I cases, 21 cases showed stage IA, 46 IB, 27 IC. We found low-(G1), moderate-(G2) and

**Table I** Correlation of cathepsin D expression in endometrial cancer with histological type, differention, myometrial invasion, and histological stage

| | *Total* | *Cathepsin D-positive* (%) | *P-value* |
|---|---|---|---|
| Histological type | | | |
| Typical endometrioid adenocarcinoma | 93 | 34 | |
| Endometrioid adenocarcinoma with squamous differentiation | 22 | 14 | NS |
| Histological stage | | | |
| pT1A | 21 | 38 | |
| pT1B | 46 | 26 | |
| pT1C | 27 | 37 | NS |
| pT2 | 12 | 8 | |
| pT3 | 9 | 44 | |
| Histological grade | | | |
| G1 | 64 | 33 | |
| G2 | 28 | 29 | NS |
| G3 | 23 | 26 | |
| Myometrial invasion | | | |
| No myometrial invasion | 22 | 41 | |
| Inner third | 30 | 20 | NS |
| Medium third | 13 | 26 | |
| Deep third | 40 | 35 | |

NS, not significant.

high-(G3) grade tumours in 55.7%, 23.3% and 20% of the cases respectively. Thirty (26%) tumours invaded the inner third, 23 (20%) the second third and 40 (35%) the outer third of the myometrium. Twenty-two (19%) tumours were confined to the endometrium.

We found no significant correlation of age and cathepsin D expression. In cases with positive cathepsin D reaction mean age was 65.1 (s.d. 8.67, minimum 49, maximum 85) years and in the group with negative reaction mean age was 64.0 (s.d. 10.02, minimum 36, maximum 84) years.

Results of cathepsin D expression with histological type, tumour grade, myometrial invasion and histological stage are presented in Table I. Data from the univariate and multivariate survival analysis are shown in Table II). The number of premenopausal patients was too small to be considered in statistical analysis. The survival distribution function grouped by cathepsin D expression is shown in Figure 2.

### Discussion

Cathepsin D is a ubiquitous acidic protease. Three forms of the enzyme are recognised: the 52 kDa precursor or procathepsin D, the 48 kDa intermediate, and the 34 kDa mature, stable form of the enzyme. In normal epithelium of mammary glands negligible amounts of the two precursor molecules accumulate intracellularly or are secreted (Nazeer et al., 1992). In human breast cancer cell lines intracellular procathepsin D processing is delayed and secretion is markedly increased. Simultaneously the two precursor molecules accumulate in the cells (Rochefort et al., 1989; Nazeer et al., 1992). In breast cancer cells cathepsin D is directly regulated at the mRNA level by oestrogen and growth factors. It is also produced in oestrogen-responsive endometrial cancer cell lines, but is not regulated by oestrogen, although it is also responsive to growth factors (Rochefort et al., 1989; Touitou et al., 1989). In the human endometrium higher cytosolic levels of cathepsin D are recognised during the luteal phase than in the follicular phase of the menstrual cycle. This fact indicates that cathepsin D is regulated in the endometrium by progesterone, without any effect of oestrogen (Maudelonde et al., 1990). In endometrial tissue cathepsin D is mainly concentrated in the glandular epithelium. Although progesterone is absent in endometrial adenocarcinoma of postmenopausal women, mean cytosolic concentration of cathepsin D is significantly higher in comparison with normal endometrium during the follicular phase. The enhanced cathepsin D level may result from the higher density of epithelial cells in endometrial adenocarcinoma (Maudelonde et al., 1990; Nazeer et al., 1994).

In patients with endometrial carcinoma involvement of pelvic or para-aortic lymph nodes is an important prognostic factor (Lurain et al., 1991). A recent study proves the correlation of cathepsin D and lymph node metastasis in endometrial carcinoma (Nazeer et al., 1994). Cathepsin D expression correlates with standard prognostic indicators of endometrial carcinoma such as tumour differentiation and myometrial invasiveness (Maudelonde et al., 1990; Nazeer et al., 1992). Unfortunately, we could not establish a correlation with the nodal status as surgical lymph node staging was not performed on a regular basis.

There is a current discussion about the value of cathepsin D as a prognostic marker (Cardiff, 1994). Some studies prove high-level cathepsin D expression as an important factor for overall survival in patients with node-negative breast cancer (Tandon et al., 1990). Cathepsin D is also a well-known prognostic factor for lymph node metastasis in breast cancer (Spyratos et al., 1989). Other authors do not suppport the prognostic value of cathepsin D or demonstrate controversial results (Henry et al., 1990; Castiglioni et al., 1994). Our study is the first to show a prognostic value for cathepsin D in endometrial cancer in a multivariate analysis.

Roger et al. (1994) have reported that the outcome of immunohistochemical staining for cathepsin D correlates with the cytosolic assay, being a valuable independent variable that is useful in estimating prognosis. The difference between cytosolic assays of whole tissue samples and the immunohistochemical analyses can be due to the relative contribution of the non-tumour cells. The biochemical method includes the total cathepsin D content of all cells and stroma, whereas the immunohistochemical method measures tumour cell enzyme content alone (Rochefort, 1992; Castiglioni et al., 1994; Roger et al., 1994). In this sense immunohistochemical detection is more specific for the tumour tissue. On the other hand immunohistochemical preparation techniques and reagents are not standardised between the different studies and different antibodies have been used in former investigations (Cardiff, 1994). Standardised immunohistochemical techniques on well-defined antibodies are important in providing reliable prognostic interpretations of cathepsin D in tumours (Cardiff, 1994). In our study we used the same antibody and immunohistochemical preparation technique as described in a recent comparative immunohistochemical and cytosolic assay study on endometrial cancer (Nazeer et al., 1994). They suggested immunohistochemically detected cathepsin D as a possible predictive factor. In our study immunohistochemically detected cathepsin D expression in endometrial adenocarcinoma showed a significant prognostic value for overall survival. Patients with tumours expressing cathepsin D had

**Table II** Cathepsin D in endometrial cancer. Overall survival analysis: univariate and multivariate analysis using the generalised Cox models including pathological stage and histological grade together with the candidate cathepsin D

| | *Univariate analysis* P-*value* | *Multivariate analysis* *RR* | *(95% CI)*[a] | P-*value* |
|---|---|---|---|---|
| Histological stage | 0.06 | 2.5 | (0.66–9.56) | 0.05 |
| Histological grade | 0.10 | 1.4 | (0.49–4.01) | 0.27 |
| Cathepsin D expression | 0.007 | 3.8 | (1.40–10.03) | 0.01 |

[a] RR (95% CI), estimated relative risk (95% confidence interval).

**Figure 2** Kaplan–Meier survival analysis of patients with cathepsin D-positive (▬) tissue expression compared with patients with a negative (——) result.

a significantly worse survival probability (Figure 2). In the multivariate analysis the association of cathepsin D with an unfavourable prognosis stayed significant.

If the association with the probability of lymph node metastasis could be established in further studies then cathepsin D could aid in decision making, especially in cases without initial surgical staging. As we found a significantly worse prognosis in endometrial cancer in cases with cathepsin D expression immunohistochemical detection of cathepsin D in a reliable standardised method could aid in predicting prognosis and planning therapy for patients with endometrial adenocarcinoma.

**Acknowledgement**
This study was supported by a Research Grant from the Mayor of Vienna (no. 1045) to Dr Kainz.

### References

BALTZER J, LAKE KJ, KUERZEL R, SCHEER KP AND ZANDER J. (1983). Prognostic criteria in patients with endometrial cancer. *Arch. Gynecol.*, **234**, 121-129.

CARDIFF RD. (1994). Cathepsin D in breast cancer: Useful? *Hum. Pathol.*, **25**, 847-848.

CASTIGLIONI T, MERINO MJ, ELSNER B, LAH TT, SLOANE BF AND EMMERT BUCK MR. (1994). Immunohistochemical analysis of cathepsins D, B and L in human breast cancer. *Hum. Pathol.*, **25**, 857-862.

CHRISTOPHERSON WM, CONNELLY PJ AND ALBERHASKY RC. (1983). An analysis of prognosticators in patients with favorable subtypes and stage I disease. *Cancer*, **51**, 1705-1709.

CLEMENT PB AND SCULLY RE. (1993). Endometrial hyperplasia and carcinoma. In *Tumors and Tumor-like Lesions of the Uterine Corpus and Cervix*, Clement PB and Young RH. (eds.) pp. 264-294. Churchill Livingstone: New York.

COX DR. (1972). Regression models and life tables (with discussion). *J. R. Stat. Soc.*, **34**, 187-209.

CREASMAN WT. (1992). Adenocarcinoma of the uterine corpus. *Curr. Opin. Obstet. Gynecol.*, **5**, 80-83.

CREASMAN WT. (1993). Prognostic significance of hormone receptors in endometrial cancer. *Cancer*, **71**, 1467-1470.

FIGO. (1990). Changes in gynecologic staging by the International Federation of Gynecology and Obstetrics. *Am. J. Obstet. Gynecol.*, **162**, 610-611.

HENDRICSON M, ROSS J, PATRICIA E, ALVARO M AND KEMPSON R. (1982). Uterine papillary serous carcinoma: A high malignant form of endometrial carcinoma. *Am. J. Surg. Pathol.*, **6**, 93-108.

HENRY JA, McCARTHY AL, WESTLEY BR, MAY FEB, NICHOLSON S, CAIRNS J, HARRIS AL AND HORNE CHW. (1990). Prognostic significance of estrogen-regulated protein cathepsin D in breast cancer. *Cancer*, **65**, 265-271.

KAPLAN EL AND MEIER P. (1958). Nonparametric estimation from incomplete observations. *J. R. Stat. Soc.*, **53**, 457-458.

LURAIN JR, RICE BL, RADEMAKER AW, POGGENSEE LE, SCHINK JC AND MILLER DS. (1991). Prognostic factors associated with recurrence in clinical stage I adenocarcinoma of the endometrium. *Obstet. Gynecol.*, **78**, 63-68.

MAUDELONDE T, MARTINEZ P, BROUILLET JP, LAFFARGUE F, PAGES A AND ROCHEFORT H. (1990). Cathepsin D in human endometrium: induction by progesterone and potential value as a tumor marker. *J. Clin. Endocrinol. Metab.*, **70**, 115-121.

NAZEER T, MALFETANO JH, ROSANO TG AND ROSS JR. (1992). Correlation of tumor cytosol cathepsin D with differentiation and invasiveness of endometrial adenocarcinoma. *Am. J. Clin. Pathol.*, **6**, 764-769.

NAZEER T, CHURCH K, AMATO C, AMBROS RA, ROSANO TG, MALFETANO JH AND ROSS JS. (1994). Comperativ quantitativ immunohistochemical and immunoradiometric determination of cathepsin D in endometrial adenocarcinoma: Predictors of tumor aggressiveness. *Mod. Pathol.*, **7**, 469-474.

NG ABP AND REEGAN JM. (1970). Incidence and prognosis of endometrial carcinoma by histologic grade and extent. *Obstet. Gynecol.*, **35**, 437-443.

ROCHEFORT H. (1992). Biological and clinical significance of cathepsin D in breast cancer. *Acta Oncol.* **31**, 125-130.

ROCHEFORT H, CAPONY F AND GARCIA M. (1987). Estrogen induced lysosomal protease secreted by breast cancer cells: A role in carcinogenesis? *J. Cell. Biochem.*, **35**, 17-29.

ROCHEFORT H, CAVAILLES V, AUGEREAU P, MAUDELONDE T AND TOUITOU I. (1989). Overexpression and hormonal regulation of procathepsin D in mammary and endometrial cancer. *J. Steroid Biochem.*, **34**, 177-182.

ROGER P, MONTCOURRIER P, MAUDELONDE T, BROUILLET JP, PAGES A, LAFFARGUE F AND ROCHEFORT H. (1994). Cathepsin D immunostaining in parafin-embedded breast cancer cells and macrophages: Correlation with cytosolic assay. *Hum. Pathol.*, **25**, 863-871.

SCHEMPER M. (1992). Cox analysis of survival data with non-proportional hazard functions. *Statistican*, **41**, 455-465.

SILVERBERG SG AND KURMAN RJ. (1992). *Tumors of the Uterine Corpus and Gestational Trophoblastic Disease*. (ed.) AFIP: Washington DC.

SPYRATOS F, BROUILLET J, DEFRENNE A, HACENE K AND ROUSESSE J. (1989). Cathepsin D: An independent prognostic factor for metastasies of breast cancer. *Lancet*, **2**, 1115-1118.

TANDON AK, CLARK GM, CHAMNESS GC, CHIRGWIN JM AND McGUIRE WL. (1990). Cathepsin D and prognosis in breast cancer. *N. Engl. J. Med.*, **322**, 297-302.

THORPE SM, ROCHEFORT H, GARCIA M, FREISS G, CHRISTENSEN IJ, KHALAF S, PAOLUCCI F, PAU B, RASMUSSEN BB AND ROSE C. (1989). Association between high concentration of Mr 52,000 Cathepsin D and poor prognosis in primary breast cancer. *Cancer Res.*, **49**, 6008-6014.

TOUITOU I, CAVAILLES V, GARCIA M, DEFRENNE A AND ROCHEFORT H. (1989). Differential regulation of cathepsin D by sex steroids in mammary cancer and uterine cells. *Mol. Cell. Endocrinol.*, **66**, 231-238.